# The Matron's Department

**Published:** 1905-09-16

**Authors:** 


### HOSPITAL ADMINISTRATION.

### CONSTRUCTION AND ECONOMICS.

### THE MATRON'S DEPARTMENT.

#### V. THE WARD SISTER.

It is hardly too much to say that the "tone" of the nurses throughout the hospital depends entirely upon the relations which subsist between the matron and the ward sisters. It is not a question of personal influence. The magnetic power possessed by some women which attracts persons of their own sex to desire passionately their good opinion, may appear to an outsider to save a great deal of trouble, and indeed some who are conscious of conspicuously lacking this gift, are tempted to despair of their powers of government. But to ensure a good tone the methods of the "magnetic" people must follow exactly the same lines as the methods of ordinary folk, or the result will be favouritism, dissension, and general demoralisation. The essential thing for the matron to obtain from her sisters is their confidence. She must depend on their reports to a great extent for her knowledge of what is passing in the hospital and without this intimate knowledge she cannot govern well. To secure the confidence of her sisters the matron must before all things else prove herself reticent. The relief of sharing heavy responsibilities with one even a little better fitted to cope with them will prove irresistible to her sisters unless experience show them that every unguarded word spoken to her makes mischief. The ward sister should be able to impart her anxieties to her matron with the certainty that she is speaking into a sealed book, that her confidences will not work round to her again through another source, that no needless fuss will arise from recounting a harmless incident. The matron who has aquired this difficult art of reticence need not fear that the confidence reposed in her by her sisters will degenerate into gossip. On the contrary it will prove the greatest safeguard against that curse of institution life indiscriminate personal "talk." For the matron will become as it were the safety valve whereby the "on dits" may be let off and the consciousness that matron knows and is silent imposes the best restraint on thoughtless tongues.

The difficulty of getting and retaining good ward sisters appears to be increasing every year, and is felt in large as well as small hospitals, in London and in the provinces alike. There is no doubt that a very happy spirit of loyalty to the institution and joy in the highest aspects of the work, rests on the nurses in those hospitals where the sisters are practically fixtures, passing 10, 20, even 30 years within its walls. It is largely within the power of the matron to create round her subordinates this atmosphere of contentment in work which is one of the best things life has to offer. How can she best do this, and at the same time keep the nursing up to the highest standard?

In the first place she must remove unnecessary worries. She must put herself hour by hour on paper through the day's work of each sister, and so organise the work that needless "drive" is abolished. She must herself supervise the sisters' quarters and take pains to make their private rooms places where the soul may be possessed in peace in off-duty hours. A comfortable bed sitting-room, if no better can be done, is a necessity if the sisters are to be more than birds of passage. An armchair, a writing table, a book shelf, a shaded light and solitude, these are the simple luxuries which should be accessible to every sister as a relief from the strain of hospital routine. And as comfort is a matter of detail we would urge on the matron the special importance of the reading-lamp. The inadequacy of the lighting arrangements in the common sitting-rooms of nurses' homes occasions far more active discomfort than shabby furniture.

The foundation being laid of contentment with surroundings and confidence established between sister and matron, there still remains the problem of keeping sisters and their subordinate nurses alike "up to their work." And to this end systematic inspection is the only road. The young sister fresh from her training school, full of self-confidence, persuaded that salvation lies only in the methods she has been taught, and the old sister who has seen things done in so many ways that she thinks nothing matters very much, both must be brought to feel the guiding hand of the matron, and both probably start with the impression that they know a good deal better what they are about than she does. The new matron will have many difficulties to face before her staff is well in hand, but she will be wise if from the very first she establishes a system of regular and open inspection into every detail of ward work. Regularity is essential, for spasmodic or furtive visitations invariably constitute a grievance. Yet the moment of inspection should be uncertain. Until the matron is sure of her own memory and powers of observation, she should make private memoranda of all the details to be looked into and assign times in each ward (different every week) for satisfying herself that her orders in this particular have been carried out. It is not to be supposed that incessant fault-finding will be the result. The mere fact that a breach of orders or discipline has been observed by the matron will generally prove sufficient. But in cases of reluctant obedience or repeated neglect, the matron, however distasteful the task, must observe the great rule of government and complain (in private) *to* the offender, never of her. It is only the incompetent ruler who is heard to say, "I wish she would not do that." The good ruler says, "Please do not do that." And how different the result!

There are three sisters who hold a somewhat different position from the others—namely, the assistant matron, the home sister, and the housekeeping sister. According to the size of the household, there may be one or other of these, if not all three. In small hospitals it is usual to delegate some of their duties to one of the ward sisters who is responsible in the matron's absence. It is a wise plan for the single-handed matron so to train her sisters that she can always rely upon one who understands the details of her office work, one who can take charge of the nurses' home and one who can give out the stores and order the meals if necessary. But of this more must be said later on.

In small modern hospitals with regular wards the number of sisters required is the same as the number of the wards, with an additional out-patient and theatre sister, and even these duties are sometimes undertaken in rotation. The staff nurse in such hospitals takes the place of the sister in off-duty times and holidays. In large hospitals the matron will find extra sisters necessary or the routine of the wards will be subject to too frequent interruptions. Skill in dove-tailing leave, and planning the duties of the sisters tells for much when the average number of beds is reckoned out among the working staff, and an experienced matron learns less and less to depend on surplus nurses.

The following is the average of available beds which falls to each sister in some of the leading London hospitals. The divergence is largely a question of construction:—

| | | | |
|---|---|---|---|
| Charing Cross Hospital ... | 16 | Great Northern Central ... | 13 |
| Guy's ... ... ... | 26 | King's ... ... ... | 22 |
| Homœopathic ... ... | 17 | London Temperance ... | 17 |
| The Middlesex ... ... | 31 | The Royal Free ... ... | 15 |
| St. Bartholomew's ... | 32 | St. George's ... ... | 15 |
| St. Mary's... ... ... | 21 | The Seamen's ... ... | 33 |
| University College ... | 14 | West London ... ... | 19 |
| The Westminster ... | 18 | | |

One word in conclusion concerning the attitude of the newly-appointed matron to the staff of sisters she finds in charge. The power of appreciating the work of a predecessor is an unerring sign of a strong character, and never is it put to a more crucial test than when a new work has to be carried through with instruments shaped by another. If the preceding influence, however old-fashioned, has been a strong and wholesome one, there will be resistance to change, but loyalty and esteem will be gained in the end if only the new comer can guard her tongue from depreciation. Let her make changes fearlessly, they will not be resented for long. But the disparaging word, the contemptuous gesture in referring to the past *régime* will never be forgotten. It is easy enough for any new matron to bring about the resignation of such sisters as are not of her own appointing. It is a higher thing to attract their adherence to the new ways.

